# A Visualization Tool for Cryo-EM Protein Validation with an Unsupervised Machine Learning Model in Chimera Platform

**DOI:** 10.3390/medicines6030086

**Published:** 2019-08-06

**Authors:** Lin Chen, Brandon Baker, Eduardo Santos, Michell Sheep, Darius Daftarian

**Affiliations:** 1 Department of Computer Science, Valdosta State University, Valdosta, GA 31693, USA; 2 Department of Natural Science, Elizabeth City State University, Elizabeth City, NC 27909, USA; 3 Department of Mathematics & Computer Science, Elizabeth City State University, Elizabeth City, NC 27909, USA

**Keywords:** protein, cryo-electron microscopy, validation, machine learning, Chimera, statistics

## Abstract

**Background:** Cryo-electron microscopy (cryo-EM) has become a major technique for protein structure determination. However, due to the low quality of cryo-EM density maps, many protein structures derived from cryo-EM contain outliers introduced during the modeling process. The current protein model validation system lacks identification features for cryo-EM proteins making it not enough to identify outliers in cryo-EM proteins. **Methods:** This study introduces an efficient unsupervised outlier detection model for validating protein models built from cryo-EM technique. The current model uses a high-resolution X-ray dataset (<1.5 Å) as the reference dataset. The distal block distance, side-chain length, phi, psi, and first chi angle of the residues in the reference dataset are collected and saved as a database of the histogram-based outlier score (HBOS). The HBOS value of the residues in target cryo-EM proteins can be read from this HBOS database. **Results:** Protein residues with a HBOS value greater than ten are labeled as outliers by default. Four datasets containing proteins derived from cryo-EM density maps were tested with this probabilistic anomaly detection model. **Conclusions:** According to the proposed model, a visualization assistant tool was designed for Chimera, a protein visualization platform.

## 1. Introduction

Cryo-electron microscopy (cryo-EM) is becoming an essential method for producing three-dimensional atomic structures of proteins due to the application of image-processing techniques [1,2,3,4] in protein modeling, as well as, the introduction of direct electron detectors [5,6,7]. Cryo-EM can help obtain protein structures in near-native conditions by freezing a macromolecule solution [8], without the concerns of crystallization in X-ray or high-energy damage in NMR. These features attract biologists to use cryo-EM, overcoming issues where X-ray and NMR struggle [9]. As of 28 July 2019, the Research Collaboratory for Structural Bioinformatics (RCSB) Protein Data Bank (PDB) [10] contains 3528 proteins solved by EM techniques, which is about 2% of the 154,243 structures on RCSB PDB. The yearly deposition number rose from 11 in 2000 to 846 in 2018 [11]. However, due to the absence of validation tools for protein structures built from cryo-EM, the cryo-EM proteins and the X-ray proteins deposited in the PDB have divergencies in the distributions of geometrical features [12]. Precautions need to be taken to avoid models containing suspicious conformations [13] assumed to be correct and used for further research [14,15].

Creating the standard criteria for assessing protein models derived from cryo-EM data is still a challenge. The Protein Data Bank community convened three Validation Task Forces (VTF) to develop standards, formats, and specifications for proteins from X-ray [14], NMR [16], and EM [17] respectively. The EM VTF report published in 2012 summarized suggestions from EM VTF participants [17]. The report recommended validating both the cryo-EM density maps and their derived models. Validation of EM density maps consists of establishing standards for assessing image resolution [18], and reporting visible structural features by the claimed resolution [19]. The validation of models involves creating criteria for assessing conformation without regard of the density maps [20], assessing a model concerning the density maps, and accessing additional data. EM VTF recommended for the development of the corresponding software [2].

The resolution is not the single factor in judging the quality of protein structures [15]. Although both X-ray and cryo-EM build protein models from their electron density maps, they have different sample preparation, different data collection, and different data processing. The resolution of X-ray proteins depends on the highest resolvable diffraction spots, whereas the definition of the resolution for cryo-EM is less clear. It is measured by Fourier Shell Correlation (FSC) from two halves of the data [21], which strives to correlate with the X-ray definition. In a cryo-EM map, various resolutions exist for areas with different fitness [22] in which the best regional resolution is usually reported in the publications. Some resolution claims cannot to be substantiated due to overfitting data [23]. It was found that the bias was not only from the software but also from the experience of the software users [15]. Regardless of these concerns, the resolution in cryo-EM data is still a factor of quality measurement of cryo-EM proteins. Though searching cryo-EM proteins by resolution was removed from RCSB PDB in early 2018, the resolution of each protein deposited is presented on its information webpage and xml metadata [24]. In a cryo-EM density map with 3 Å resolution or better [25,26,27,28], the conformations of sidechains are generally distinguishable. It is an open question of how accurate cryo-EM structures are, particularly for those derived from the highest-quality cryo-EM density maps.

The current validation system for cryo-EM proteins in PDB borrows the tools for X-ray crystallography. Since the early 1990s, many validation tools have been developed for X-ray proteins to address various aspects such as validation of experimental data, protein models, and the fit between experimental data and protein models [29,30,31,32,33,34]. The structure determination process of cryo-EM proteins is similar to X-ray proteins except for the collection and processing of the experimental data. The wwPDB Deposition & Annotation (D&A) system used at RCSB PDB before 2018, designed for X-ray proteins, was also used to validate NMR proteins and cryo-EM proteins [35]. At that time, three types of validation reports at RCSB PDB were organized in one category, and the metrics for X-ray proteins were applied for NMR proteins and EM proteins. At the end of 2017, the OneDep validation system [36] was launched. The validation reports are now divided into three categories for X-ray, NMR, and cryo-EM respectively at PDB, the three types of reports use different validation metrics. The quality metric values in EM validation reports are calculated by MolProbity [37,38]. A residue compares its bond, angle, torsion angle, and contact distances of a residue with the ideal values [36]. A residue is colored green if no outliers are detected, yellow if containing one type of outlier, orange if two types of outliers, red if there are three or more types of outliers. Note that the outliers may not represent errors in the model. Instead, those outliers may be genuine and unusual, which may be of biological interest. In order to account for the changes in protein quality, the validation reports are regenerated annually for the entire public archive.

In the previous study [39], we observed that geometrical features from the cryo-EM structures have a different distribution to the features from X-ray structures. The difference is potentially due to the miss-detection of conformation errors by the current validation tools in the OneDep system [36], which was designed for X-ray protein structures. In the current study, a protein validation tool with graphical user interface (GUI) was designed to implement the proposed unsupervised model [39]. The tool serves as the complementary for the validation pipeline of the OneDep system and is embedded in the University of California San Francisco (UCSF) Chimera platform [40] for the interactive validation. UCSF Chimera is a highly extensible visualization platform for interactive visualization and analysis for proteins and cryo-EM density maps. With the support of the Chimera Application Programming Interface (APIs) [41], protein researchers have been developing plugins for the Chimera system according to their research for the past decade [42,43,44,45]. These third-party plugins and the Chimera built-in plugins help users to visualize three-dimensional protein conformations, cryo-EM density maps, and extract needed information. Tkinter and Biopython were used to build the GUI and the unsupervised model. Tkinter is a graphic design Python module, and Biopython is a computational biology open-source Python module [46].

## 2. Materials and Methods

One high-resolution X-ray data set was used as the reference data set. The data set, referred to as X-ray-1.5, contains 9131 protein structures that are solved with X-ray crystallography and have a resolution better than or equal to 1.5 Å. At the resolution 1.5 Å, the atoms in a protein can be located precisely. Therefore, we assume the conformation errors in X-ray-1.5 are rare and feasible to be used as the reference data set. The protein structural data in X-ray-1.5 was downloaded from the RCSB PDB website [10] in March 2018, with sequence similarity less than 90%. In order to avoid the effects of non-crystallographic symmetry [47], chains with 95% sequence similarity with any other chains in each protein were ignored.

Four high-resolution EM datasets were used to analyze the conformations of the proteins solved with Cryo-EM and to choose the default cutoff value for the proposed unsupervised model. One EM data set containing proteins solved from cryo-EM density maps with a resolution between 2 and 4 Å before 2017, referred to as EM-2-4-2016, has 215 proteins. The second EM data set contains 163 EM proteins solved before 2017 with a resolution between 4 and 6 Å, is referred to as EM-4-6-2016. The third EM data set is referred to as EM-2-4-2018, in which there are 288 EM proteins solved from 2017 to March 2018 with a resolution between 2 and 4 Å. The fourth EM dataset, referred to as EM-4-6-2018, has 160 EM proteins solved from 2017 to March 2018 with resolutions between 4 and 6 Å. The proteins in EM-2-4-2016 and EM-4-6-2016 were downloaded in December 2016, and the proteins in EM-2-4-2018 and EM-2-4-2018 were downloaded in March 2018 from RCSB PDB [10].

**Figure 1:**
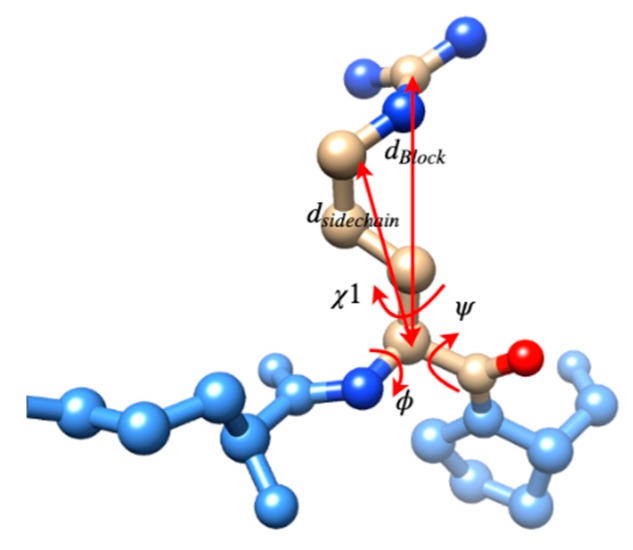
The five selected conformation features for protein residues, φ, ψ, $\chi_{1}$, $d_{sidechain},$ and $d_{block}$.

Five features, backbone torsion angle Phi (φ) and Psi (ψ), sidechain torsion angle ($\chi_{1}$), sidechain size ($d_{sidechain}$), and block length ($d_{block}$), were selected to describe the conformation of a protein residue as shown in Figure 1. The torsional angle φ is formed by atom C located in the previous residue of the protein sequence and atoms N, CA, C in the target residue. N, CA, C are the atom names in a residue representing the nitrogen atom, the carbon atom connecting to a sidechain and the carbon atom in the carbonyl along the protein backbone from the N side to C side. The angle ψ is formed by atoms N, CA, C in the target residue and atom N in the next residue in the sequence. The dihedral angle $\chi_{1}$ is the first torsion angle in the sidechain which is formed by atoms N, CA on the backbone and CB, and CG on the sidechain. The range of torsion angles in the study is 0° to 360°, which is from Biopython directly instead of −180° to +180° in the Ramachandran plot [48]. Eighteen of 20 residues were used since glycine (GLY) and alanine (ALA) have no $\chi_{1}$ due to their small size of side-chains. $d_{sidechain}$ is the distance between the CA atom on the backbone and the mass centroid of the sidechain atoms. $d_{block}$ is the distance between the CA atom on the backbone and the mass centroid of the distal block of a specific residue. The blocks of the residues were defined in He’s study [49].

For each of 18 types of residues, normalized probability density functions (npdfs) were calculated for the five selected features using X-ray-1.5. In each protein structural file, the residues in the chains with less than 95% sequence similarity were selected. The values of the five features were calculated from the selected residues. The corresponding pdfs were plotted with MATLAB for the five features of each residue type. The bin size of 5° for ϕ, φ, and $\chi_{1}$ and 0.05 Å for $d_{sidechain}$ and $d_{block}$ were used for calculating the pdf. Total 90 (18 × 5) pdfs were generated. Each pdf plot was then normalized with its highest peak value. The normalized plots are referred to as normalized pdfs (npdfs).

The Histogram-Based Outlier Score (HBOS) values of each residue were calculated for the five features from their npdfs, according to Equation (1). For example, for a residue, its HBOS(i) is log(1/npdf_i_(v_i_)) in which npdf_i_(v_i_) is the value read from the npdf plot of its residue type generated from X-ray-1.5 with feature value v_i,_ i is the feature index. If the value of $npdf_{i}$ is less than 0.001, the HBOS(i) value in that bin was assigned a value of 5 to avoid an infinite HBOS value. A high HBOS above 2 means the feature value is highly unfavorable. The HBOS score of a residue is the summation of the five HBOS values from its five features. The lower the HBOS score, the more preferred is the conformation.

(1) $$HBOS=\overset{5}{\underset{i=1}{\sum }}HBOS\left(i\right)=\overset{5}{\underset{i=1}{\sum }}\log\left(\frac{1}{npdf_{i}\left(v_{i}\right)}\right)$$

**Figure 2:**
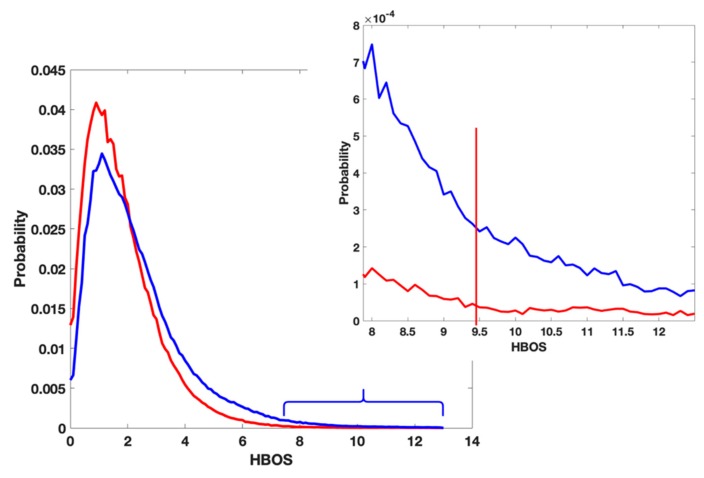
The distribution of Histogram-Based Outlier Score (HBOS) value of residue in X-Ray-1.5 (red) and Electron Microscopy (EM) data sets (blue).

The threshold of labeling a residue as an anomaly was chosen from the histogram plots of the HBOS values shown in Figure 2. The HBOS values of the residues in X-ray-1.5 after removing non-crystallographic symmetrical chains were calculated with Equation (1). The probability density function of the HBOS values is plotted (red) in Figure 2. A similar plot was generated from the residues in the four EM data sets. The corresponding probability density function is plotted (blue) in Figure 2. The bin size of the two plots is 0.1. The probability of having HBOS value above 9.5 is near to zero in the X-ray plot (the zoomed plot). However, there are plenty of EM residues having HBOS values above 9.5. Due to the high resolution of X-ray-1.5, we assume that the protein models in X-ray-1.5 are the correct conformations. We took the HBOS plot of X-ray-1.5 (red) in Figure 2 as the ground-truth HBOS distribution. Compared with the plot of X-ray-1.5, it is reasonable to label a residue in the EM datasets as an anomaly if its HBOS value is above 9.5. To maintain high confidence, the HBOS value 10 was chosen as the default threshold in the visualization tool. The residues having HBOS values above 10 in the EM data sets were labeled as anomalous residues.

**Figure 3:**
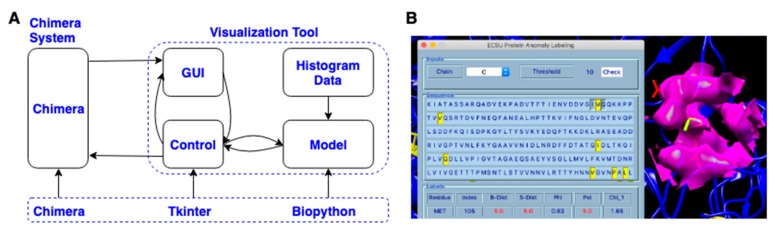
(**A**) The architecture of the visualization tool; (**B**) The Graphical User Interface (GUI) of the tool in Chimera.

A visualization tool in Chimera using the proposed unsupervised model was designed and delivered. The code of the visualization tool was organized with the Model-View-Controller (MVC) model. As shown in Figure 3A, the visualization tool contains a GUI, control code, model code, and histogram data generated from X-ray-1.5. The protein loaded in the Chimera platform can be accessed by both GUI and the control model. The GUI coded with the Python Tkinter module has input and output widgets. As shown in the top section of the GUI in Figure 3B, the input widgets receive chain ID and threshold values from the user. These input values are then passed to the control module by clicking the “Check” button. The control module uses the received values as parameters to calculate HBOS scores for each residue in the selected protein chain. The model loads the pre-generated npdf data and identifies suspicious residues with the entered thresholds. These suspicious residues are highlighted with yellow color and displayed in both the middle section of the GUI and the Chimera major window (black background) by the control model (Figure 3B). The tool was coded mainly in three Python modules: Chimera, Tkinter, and Biopython. The Chimera platform is an isolated protein visualization system from the default Python distribution which contains the Chimera module and Tkinter module by default. The Chimera module provides APIs to access and manipulate protein data loaded in the Chimera software. Tkinter is one of the GUI modules in the Python community used to support visualization tools written by Chimera engineers and third-party research groups [40]. Biopython module [50] was used to extract protein geometrical features, generate npdfs, and calculate chain similarity [46]. The tool has been tested in Chimera 1.13.1, which can be downloaded from the Chimera official website [51].

To label anomalous residues of a loaded protein in Chimera with the designed tool, load the target protein from Chimera by clicking “open”, then click “Tools- > Utilities- > ECSU Label” to start the tool. The popup GUI of the tool has three sections: inputs, sequence, and labels from top to bottom (left in Figure 3B). Users can choose the chain and input the threshold value in the inputs section. After choosing input arguments, click the “Check” button to run the unsupervised machine learning label code. The sequence section shows the residue sequence of the selected chain, in which each residue is represented with its single-letter name. The identified anomalous residues are highlighted with yellow color in the sequence. The corresponding residues are also highlighted with yellow color with the sidechain shown in the Chimera window (right side in Figure 3B). In the label section, residue name, residue index, and HBOS scores for five features are displayed for each labeled residue. The columns “Residue”, “Index”, “B-Dist”, “S-Dist”, “Phi”, “Psi”, “Chi_1” represent the residue name, residue index, φ, ψ, $\chi_{1}$, respectively. The individual HBOS scores above 2 are highlighted with red color.

The Python scripts and MATLAB scripts of generating npdfs were deposited to Github at https://github.com/lin-chen-VA/medicines. The Python source code and installation tutorial of the visualization tool were deposited to https://github.com/lin-chen-VA/chimeraplugin.

## 3. Results

### 3.1. Cons in the Current Validation Tool

**Figure 4:**
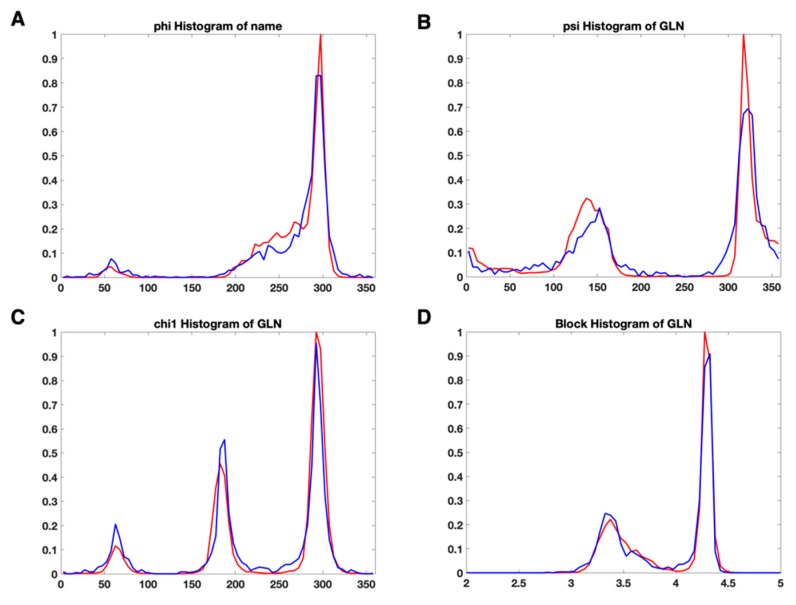
The normalized histogram plot of four features of Glutamine (GLN) for X-ray-1.5 data set (red) and EM-2-4-(2016, 2018) data set (red). (**A**) the normalized histogram plots of φ; (**B**) the normalized histogram plots of ψ; (**C**) the normalized histogram plots of $\chi_{1}$; (**D**) the normalized histogram plots of $d_{block}$.

We investigated the distribution of torsion angles (φ, ψ, $\chi_{1}$), $d_{sidechain}$, and $d_{block}$ for each residue type. There were no significant differences observed between the plot from the X-ray dataset and the plot from EM datasets. Figure 4 shows the normalized histogram plots of φ (Figure 4A), ψ (Figure 4B), $\chi_{1}$ (Figure 4C), and $d_{block}$ (Figure 4D) for Glutamine (GLN). In each part of Figure 4A–D, the red plot represents the histogram distribution of the feature values for a specific feature in X-ray-1.5 dataset. The plots are normalized with the highest value in each plot. The blue plots were generated from EM-2-4-2016 and EM-2-4-2018. In order to compare with the X-ray plots, the plots from EM datasets were also normalized with the highest value in the corresponding X-ray plots. The X-ray plots and EM plots had peaks at almost the same positions. Such as, in Figure 4C, GLN has three peaks at 62.5°, 185°, and 292.5° in both the X-ray plot and the EM plot. Although sample points between 220° and 250° are observed in the EM plot, the X-ray plot has much lower probability in the same range. The similarity of the two plots reveals that the single feature is not sufficient to distinguish an anomaly from typical values. Considering the noise and the size of the dataset, the EM proteins and X-ray proteins have roughly the same distribution in Figure 4A,B,D. The same distribution characteristics are noted in the other 17 residue types (not include GLY and ALA). An individual suspicious geometrical feature value may not be a hint to identify an anomalous residue. In the current OneDep system at PDB, MolProbity validates the single feature or a few combined features step by step with a pipeline. The lack of interaction terms from different types of features gives the validation procedure high alignment with the modeling procedure. The suspicious conformations introduced from modeling are likely taken as the acceptable or even the favorable conformations.

**Figure 5:**
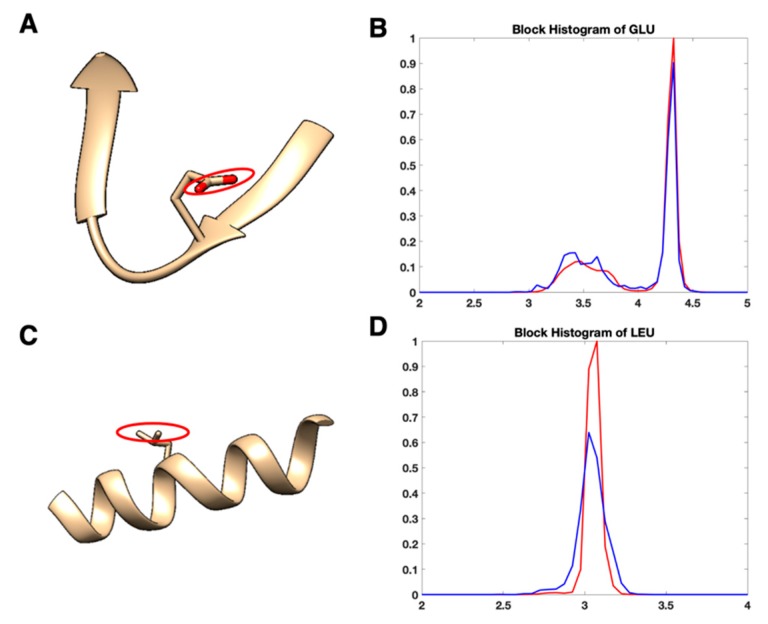
Two residues having abnormal sidechain size are labeled as safe in the Protein Data Bank (PDB) validation reports. (**A**) Glutamic acid (GLU) indexed 150 in chain C of protein 6bgo; (**B**) the histogram plot of $d_{block}$ for GLU; (**C**) Leucine (LEU) indexed 338 in chain C of protein 6b5v; (**D**) the histogram plot of $d_{block}$ for LEU.

The current validation tool in the OneDep system at PDB may contain not enough features for conformation validation. The bond, angle, torsion angle, and contact distances are majorly used as the evaluation metric. However, we noted that there are plenty of residues labeled as safe (green color in the validation reports) containing abnormal conformations. Figure 5 shows two such residues. Figure 5A is Glutamine acid in the protein 6bgo, in which the tail block (circled) is bent toward the backbone excessively. Its φ, ψ, $\chi_{1}$ are 252.3°, 134.5°, and 286.0° respectively, which are all located in the favorable value zone. Thus, its conformation is labeled as safe in its PDB validation report. Nevertheless, it has an abnormal $d_{block}$ value of 2.97 Å. The probability of having a $d_{block}$ less than 3 Å is extremely low in X-ray-1.5 dataset, as shown in Figure 5B. $d_{block}$ is a feature not involved in the current PDB validation tool. This residue is a potential anomaly according to the experience of our subject matter experts. In Figure 5C, the Leucine (LEU) in chain C of protein 6b5v has an abnormal long sidechain. Its tail block (circled) is bent toward the opposite direction of the backbone. As shown in Figure 5D, the value 3.27 Å is much longer than the observed up-limit value of 3.25 Å in X-ray-1.5. These two samples demonstrate that the atoms of the tail blocks can move toward different directions by keeping the bond length and torsion angles in a favorable value range. This degree of freedom cannot be fully monitored by the current geometrical metrics in the OneDep system. We suggest introducing more indirect features in the validation tool to handle those extra degrees of freedom, such as $d_{block}$.

### 3.2. Use Combined Multi-Features

**Figure 6:**
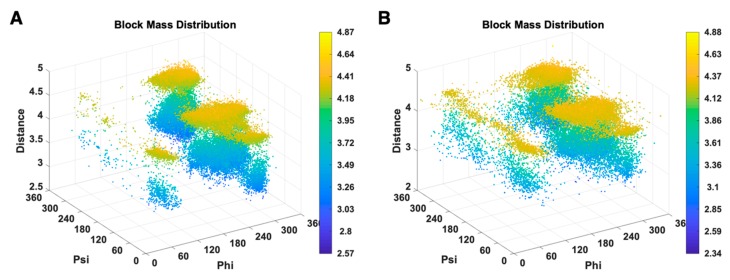
3D scatter plots of (φ, ψ, $d_{block}$) of GLU are shown for X-ray-1.5 data set (**A**), EM-2-4-(2016, 2018) data set (**B**).

We investigated the distribution of combined features (φ, ψ, $d_{block}$) of GLU for X-ray-1.5 (Figure 6A), EM-2-4-2016 and EM-2-4-2018 (Figure 6B). Each data point in the plots represent a GLU residue with a particular conformation, and points are colored according to the $d_{block}$ value, which changes from blue to yellow for the values from low to high. In Figure 6A, most of the data points are clustered within several areas. GLU plots have four large clusters in Figure 6A at (270°, 140°, 3.5 Å), (270°, 140°, 4.3 Å), (270°, 0°, 3.5 Å), (270°, 0°, 4.3 Å), and two small clusters at (60°, 50°, 3.5 Å), (60°, 50°, 4.3 Å). The data points are briefly classified into two layers at 3.5 Å and 4.3 Å of $d_{block}$ value. The GLU residues in X-ray-1.5 are rarely observed outside those areas. In Figure 6B, the data points are distributed widely in EM-2-4-2016 and EM-2-4-2018 and are less concentrated than the cluster areas in Figure 6A. The data clusters at (270°, 140°, 3.5 Å), (270°, 140°, 4.3 Å), (270°, 0°, 3.5 Å) in Figure 6B are wider and sparse compared with the clusters in Figure 6A. Especially, in the range from (60°, 0°, 4.3 Å) to (60°, 360°, 4.3 Å) and the range (60°, 0°, 3.5 Å) to (60°, 360°, 3.5 Å) in Figure 6B, the data points are uniformly distributed and form two belts. The conformations falling into these two belts have a relatively high risk of being anomalies. The distribution difference that is not distinguishable in the plots of single features is clear and distinct in Figure 6. It gives clues that the combination of different types of features is more efficient in validating protein conformations. Although the current PDB validation tool considers residue conformations as acceptable/favorable using Ramachandran criteria and rotamer libraries, the combinations of more indirect features should be used as the validation metrics. Evaluation of multi-features simultaneously instead of the current pipeline approach may improve the performance of validation tools.

### 3.3. A Complement of the Current PDB Validation Tool

**Figure 7:**
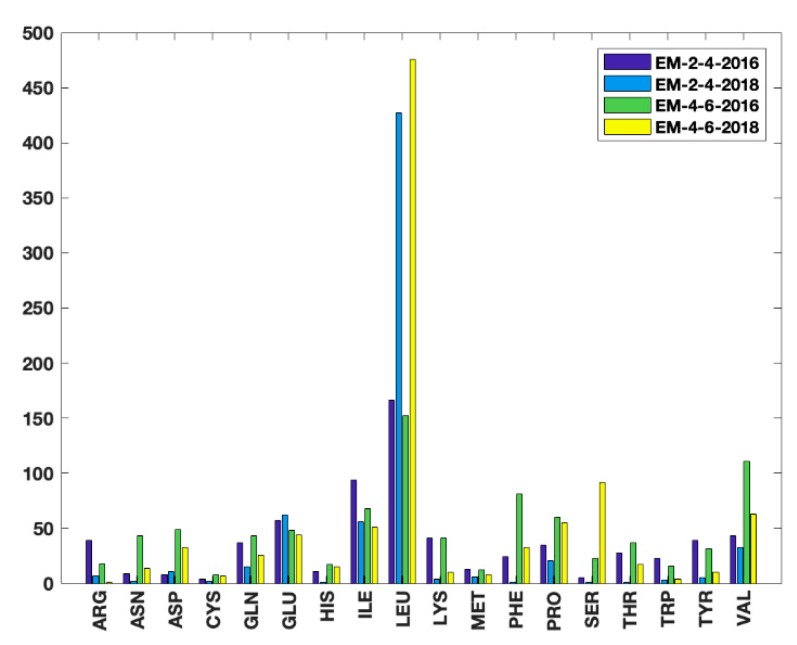
The number of 18 types of residues which are labeled as anomalies by HBOS score and safe in PDB validation reports.

The proposed unsupervised HBOS model was used to identify outlier residue conformations. The residues with HBOS value greater than 10 were labeled as the anomalous residue in four EM datasets. Two Master students majored in biology confirmed the outlier residues labeled by the HBOS model manually. The residues which are labeled safe in the PDB validation reports but identified as anomalies by the HBOS value are summarized in Figure 7. The metadata xml files of the validation reports were downloaded from the repository of the xml metadata of the protein validation reports [24] with a web crawler (deposited at https://github.com/lin-chen-VA/medicines/blob/master/labelling.py). The outlier information was fetched from the property plot section of the PDB validation reports. The metadata contains the mark of “OUTLIER” if Ramachandran outliers exist, the mark of “OUTLIER” if rotamer outliers exist, a list of clashes, a list of angle outliers, and a list of plane outliers. The validation reports give scores based on how many types of outliers in the five types of outliers have been found in the metadata. For example, if a residue has the mark of rotamer outliers and four atom clashes; it has score 2 in its validation report. Note that the validation reports do not quantitatively report how extreme an outlier is, which means the validation reports cannot give hints of how risky it is of having a specific value. The higher score outlier may not be worse than the lower score outlier. Only five features are used in the HBOS model, which contains three geometrical features (φ, ψ, $\chi_{1}$) used in the current PDB validation tool and two proposed indirect features ($d_{sidechain}$, $d_{block}$). Thus, the HBOS model cannot label all outliers labeled in the PDB validation reports. Nevertheless, the HBOS model is often sensitive in identifying the anomalies that are not labeled by the current PDB validation tool. Before more features are introduced into the HBOS model, it is feasible to use the HBOS model as a complement to the current PDB validation tool.

The numbers of residues that are labeled as safe in PDB validation reports and anomalies by the HBOS model for 18 residue types in four EM datasets are summarized in Figure 7. Such residues are referred to as HBOS outliers. The four bars from left to right for each residue type represent the results from EM-2-4-2016, EM-2-4-2018, EM-4-6-2016, and EM-4-2018, respectively. It is to be noted that residues with a large sidechain were assigned better conformations from 2016 to 2018. For instance, 39 arginines (ARG) HBOS outliers were labeled from the 215 proteins of the EM2-4-2016 dataset. The number drops to seven in the 288 proteins of EM-2-4-2016. Only one ARG HBOS outlier ws identified from the 160 proteins in EM-4-6-2018. Considering that the mid-resolution density maps have higher uncertainties of atomic coordinates, the quality improvement in EM-4-6-2018 may be due to the use of conformation information extracted from known protein structures, by which the favorable feature values are assigned to residues. It is also noted that Leucine (LEU), Serine (SER) have much more HBOS outliers labeled in EM-4-6-2016 and EM-4-6-2018 than EM-2-4-2016 and EM-2-4-2018. Most of those LEU and SER outliers are located on long loops or loops between two secondary structures, where residues have more flexibility for assigning conformations. The unexpected results can be generated from the essential modeling process. After assigning reasonable conformations to most of the residues in the sequence, the conformation of LEU and SER on loops are assigned. At this moment, it is challenging to have LEU and SER reasonable conformations to meet all constraints. In order to meet the majority of constraints, instead of using the conformations with rigid bonds and plane angles for LEU and SER, the coordinates of the atoms on the sidechain are slightly adjusted. Although the dihedral angles are still in the favorable region, the conformations are abnormal. The current tool in the OneDep system is not sensitive enough to identify these outliers. In the HBOS model, $d_{sidechain}$ and $d_{block}$ are effective in monitoring the anomalous residue from the adjustment of the atom coordinate. By combining with the other three torsion angles, the model is sufficient to serve as a complement to the current PDB validation tool.

### 3.4. Visualization Chimera Tool

The visualization tool which labels anomalous residues on the 3D protein conformations can help structural biologists promptly verify the labeled residues, consider the effects from neighboring residues, and evaluate the fitting to cryo-EM density maps. Like the validation reports generated from the OneDep system, the HBOS model provides the geometrical information and HBOS score for labeled residues. The information is isolated from 3D structures and cannot provide enough clues directly for scientists to figure out the reasons for causing those conformational anomalies. The current visualization tool infuses both conformation validation and visualization. Users can cross-validate 1D sequence information, 3D space information, and HBOS scores within one window. It is feasible to connect information from different sources promptly.

The visualization tool was designed for the Chimera platform as an open-source utility, which used Chimera module, Tkinter module, and Biopython module. Biopython containing submodules of extracting protein information, sequence processing has been adopted for a decade in bioinformatics and computational biology research. The Chimera module built in the Chimera platform contains functions of protein structure retrieve, piece selection, deletion, highlighting, and related operations. However, Biopython and Chimera have a different data structure to save protein information. Therefore, they are not able to share data directly. The current tool uses an interface code to pass data between them. The data passing between two modules slightly increases CUP time and memory cost. Keeping the visualization features, using the Chimera protein structure only may be a solution. However, Chimera itself only supports Python 2. It means, including the current visualization tool, Chimera built-in tools, and third-party tools were written in Python 2. Since the Python community has decided to stop the support for Python 2 in 2020, ChimeraX [52] will be the next generation of Chimera. As biological research teams have been writing the tools for the Chimera platform for a quite long period, it is not feasible to move those third-party tools to Python 3. Predictably, the Chimera community will stick with Python 2 for an extended period. The current tool will be continually updated before ChimeraX is widely accepted by the protein research community.

## 4. Discussion

The current PDB protein validation in the OneDep system may not detect outlier residue conformations effectively. The validation system at PDB validates one or a few features step by step with a pipeline. The lack of some interaction terms of features in the validation model may miss labeling some outliers. The validation metrics in the current PDB validation system contain the bond, plane angles, torsion angles, and contact distances. However, it is to be noted that those metrics work in the case where the bond length and plane angles of residues are rigid. The movement of tail atoms, such as CD1 and CD2 atoms of LEU, can turn out residue outliers with abnormal sidechain size and favorable torsion angles.

The proposed unsupervised model uses the combination of five geometrical features to label residue outliers. The combination of features can improve the confidence of labeling and avoid the domination of a single feature value. $d_{sidechain}$ and $d_{block}$ describe the size and the shape of the residue sidechain to help detect the abnormal shift of side-chain tail atoms. Although the environmental factors from the neighbors of a residue and the residue interactions are not considered, the model is feasible to serve as a complement to the current PDB validation tool and help label the missed outliers in the validation reports. A GUI tool implementing the proposed HBOS model was designed for the Chimera visualization platform. The location of the 1D sequence, HBOS scores of features, and the conformation in 3D space of an outlier residue are visible to users in a single window. It is feasible to allow users to cross-validate the conformation and illustrate the possible reasons for introducing such outliers in the modeling process.

The proposed unsupervised HBOS model is a derivative of Naïve Bayes. More features usually bring better performance, even if some of the features are not independent of each other. Since labeling a residue conformation most likely depends on the subjective judgment of subject matter experts, it is challenging to have a widely accepted labeled dataset for training a supervised model. The proposed model breaks the dilemma of lack of labeled dataset required by supervised models. In order to leverage the model, more features about fitting between residue models and cryo-EM density maps, and residue interactions will be introduced into the models in further study. Highly accurate supervised models will be built based on the labeled results from the unsupervised model.
